# A Fuzzy Permutation Method for False Discovery Rate Control

**DOI:** 10.1038/srep28507

**Published:** 2016-06-22

**Authors:** Ya-Hui Yang, Wan-Yu Lin, Wen-Chung Lee

**Affiliations:** 1 Research Center for Genes, Environment and Human Health, and Institute of Epidemiology and Preventive Medicine, College of Public Health, National Taiwan University, Taipei, Taiwan

## Abstract

Biomedical researchers often encounter the large-p-small-n situations—a great number of variables are measured/recorded for only a few subjects. The authors propose a fuzzy permutation method to address the multiple testing problem for small sample size studies. The method introduces fuzziness into standard permutation analysis to produce randomized p-values, which are then converted into q-values for false discovery rate controls. Simple algebra shows that the fuzzy permutation method is at least as powerful as the standard permutation method under any alternative. Monte-Carlo simulations show that the proposed method has desirable statistical properties whether the study variables are normally or non-normally distributed. A real dataset is analyzed to illustrate its use. The proposed fuzzy permutation method is recommended for use in the large-p-small-n settings.

Biomedical studies nowadays often involve a large number of variables for the study subjects, such as gene expression data analyses and genome-wide association studies, etc.^1^,^2^,^3^,^4^. To test whether there is a difference between different groups (e.g., between breast cancer patients and healthy controls) in any of these variables, one needs to properly account for the multiple testing problem. The traditional approach to control the familywise error rate (FWER, the probability of one or more false positives among all the hypotheses) is too stringent however, especially when the number of tests is large.

Benjamini and Hochberg^5^ introduced a new error measure called the false discovery rate (FDR, the expected proportion of incorrect rejections among all rejections). Controlling FDR results in more powerful tests as compared to controlling FWER. Storey^6^ later introduced the ‘q-values’, and showed how they can be calculated from a set of p-values. We can reject the null hypothesis of no significant difference between groups for any variable with a q-value less than or equal to a certain level, say 0.05. This will control the FDR at 0.05, if the p-values for the null hypotheses are distributed according to a uniform (0, 1) distribution, and the p-values for the alternative are stochastically smaller.

In this paper, we are concerned with situations where only very few subjects (say, <10) are recruited but a great number of variables (say, >10000) are measured/recorded for each person—the ‘large-p-small-n’ situations. (For example, because of the high costs of RNA-Seq technologies, studies using the technologies are of limited sample sizes, usually *n* < 10^7^,^8^. Another example is for batch-effect testing, where a small fraction of the total samples, usually *n* < 10 again, are repeatedly tested in different batches^9^). For a sample size so small, the central limit theorem no longer applies. A parametric test, such as Student t test, therefore cannot guarantee uniform (0, 1) distributed p-values under the null. A non-parametric test, such as the Wilcoxon rank-sum test, or the standard permutation method^10^, fares no better; under the null it produces p-values that are discretely distributed (instead of being uniform and continuous within 0 and 1) for small sample size studies. While the problems of non-uniformity of the parametric tests and the discreteness of the non-parametric tests are well known, their effects on FDR control are less well recognized.

In this paper, we propose a fuzzy permutation procedure for FDR control. We add a tiny number called a fuzzy term to the p-value of each variable and then run a permutation analysis. Effectively, this produces randomized p-values that are uniformly and continuously distributed between 0 and 1 under null hypothesis. We will study the performance of the proposed method using algebra and Monte-Carlo simulations. We will also demonstrate its use with a real dataset.

## Methods

Assume that a case-control study recruits a total of *n*_1_ cases and *n*_0_ controls. A total of *m* variables (indexed by *j*) are measured for each and every subject in the study. The algorithm of the fuzzy permutation procedure is described below:

1. Perform a two-sample test, such as Student t test or Wilcoxon rank sum test, and calculate the p-value, *p*_*j*_, for *j* = 1, …, *m*.
2. Add a fuzzy term to the p-value: 
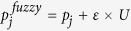
, for *j* = 1, … *m*, where *U* is a uniform (0, 1) distribution, and *ε* is a very ‘small’ number (explained later).
3. Permute case and control status, and calculate the p-value 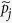, for *j* = 1, … *m*, based on the permuted data.
4. Add a fuzzy term to the p-value: 
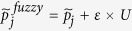
, for *j* = 1, … *m*.
5. Repeat Steps 3 and 4 for a total of *k* times.
6. Calculate a permutation-based p-value, 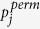, as the proportion with 
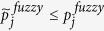
 among the total *k* permutations, for *j* = 1, … *m*.
7. Convert the permutation-based p-values in Step 6 to q-values.

Note that the fuzzy terms to be added are very small numbers. Ideally one should let *ε* → 0, so that they will have absolutely no effects on the ranking of the p values when they are not tied. But *ε* should not be exactly zero, otherwise ties will not be resolved. In practice, we can let *ε* to be slightly larger than the precision of a computer as allowed by a programming language. The limit of resolution for the widely used R code is 10^−16^. Thus one can safely use *ε* = 10^−12^, which shall be small enough for all data size. S1 Exhibit presents an alternative but equivalent algorithm based on fractional counting.

Theoretical comparisons of fuzzy and standard^10^ permutation methods are presented in S2 Exhibit, which shows that the fuzzy permutation based p-values are distributed according to a uniform (0, 1) distribution under the null, and that the fuzzy permutation method is at least as powerful as the standard permutation method under any alternative.

A function (fuzz.perm) written in R code^11^ is provided in S3 Exhibit. Input the case-control data to it as the argument, and the function will output the fuzzy permutation q-values automatically.

**Figure 1:**
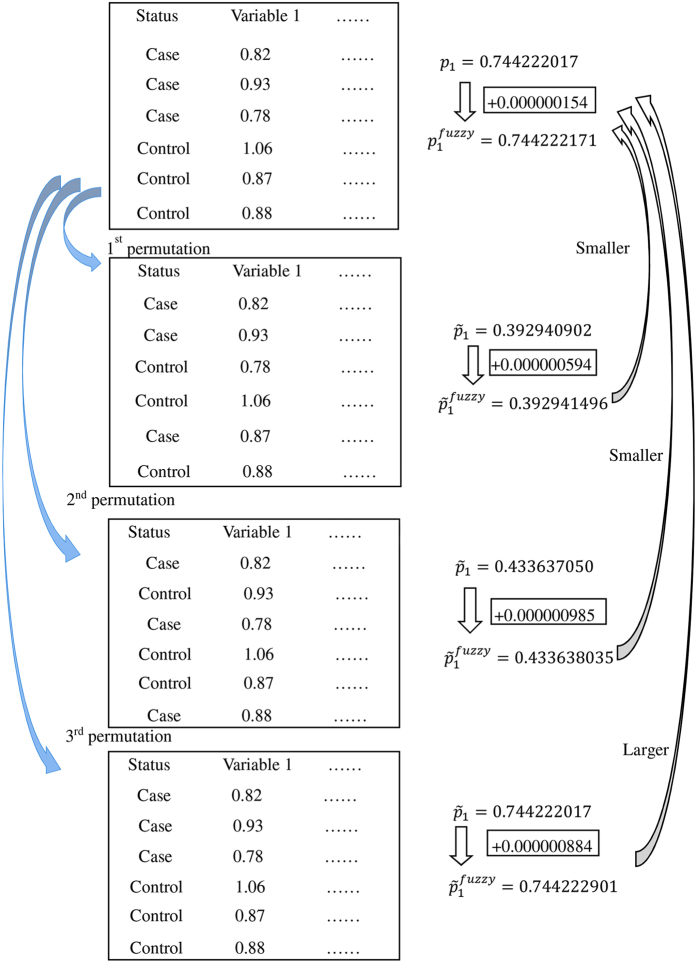
Demonstration of the fuzzy permutation algorithm. The original data and its three permutations are shown in the boxes. Four fuzzy terms (respectively for the original data and the permutations) are shown in the rectangles.

Figure 1 demonstrates the algorithm using a hypothetical case-control data. For brevity, only the first variable and the first three permutations are shown. The p-value for the variable is calculated to be *p*_1_ = 0.744222017 (using two-sample Student t test). After adding a fuzzy term (*ε* × *U* = 10^−6^ × 0.154 = 0.000000154; here we use a larger *ε* = 10^−6^ for ease of demonstration) to the p-value *p*_1_, we obtain 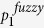 = 0.744222171. At the first permutation, the p-value is 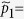
0.392940902. Another fuzzy term (0.000000594) is then added to the p-value 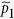 to obtain 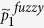 = 0.392941496, which is smaller than 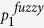
(0.744222171). At the second permutation, 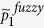 is smaller than 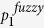
(0.433638035 < 0.744222171). At the third permutation, a tie occurs (*p*_1_ = 0.744222017 = 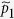). And this is resolved by the fuzzy terms: 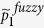 = 0.744222901 > 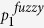. Note that the added fuzzy terms have no effects on the ranking of the p-values between the original data and the first and the second permutations.

## Results

### Simulation Study

We perform Monte-Carlo simulations to study the performances of the fuzzy permutation procedure. We assume the study variables to follow respectively, the normal distribution, the gamma distribution, the truncated normal distribution and the beta distribution. The total number of variables is set at 100000. For each of the four types of distributions, we consider four scenarios: HS, HW, LS and LW, respectively (H and L: high and low signal-to-noise ratios; S and W: strong and weak signals). The parameters for all these scenarios are detailed in S4 Exhibit. We assume a total sample size of 10 (5 cases and 5 controls).

Using the algorithm described above, we calculate the permutation-based p-values using the fuzzy permutation method with Student t test and with Wilcoxon rank sum test, respectively. A total of 5000 permutations were performed for each of the HS, HW, LS and LW alternative hypotheses. [We also simulate the null hypothesis when all the variables are noises and find that the fuzzy permutation p-values are indeed distributed according to a uniform (0, 1) distribution under the null (results not shown).] For comparison, we also calculate the p-values, using the Student t test (without permutation), the Wilcoxon rank sum test (without permutation), as well as the standard permutation method^10^ with Student t test and with Wilcoxon rank sum test, respectively.

We use Package fdrtool in R^11^ to convert the p-values to q-values. In our simulation, there are a total of 100000 variables, and therefore, a total of 100000 q-values. The q-values are ranked from the smallest to the largest. For each q-value, we calculate the false discovery proportion (FDP, the proportion of incorrect rejections among all rejections, or in our case, the proportion of null variables among those variables with q-values less than or equal to the specified q-value). We then plot FDPs against q-values. Because FDPs with small denominators are unstable, in the plot we begin with the 501th smallest q-value.

**Figure 2:**
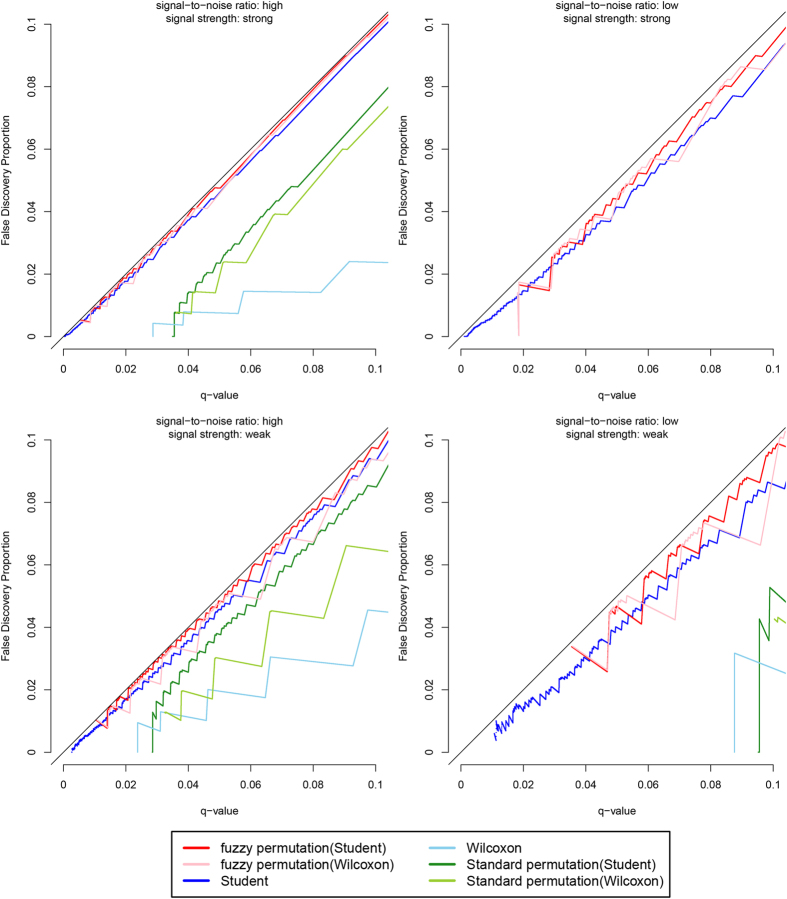
Plot of false discovery proportions against q-values, when the variables are normally distributed.

The results when the variables are normally distributed are shown in Fig. 2. It can be seen that the false discovery proportions are very close to the specified q-values for the fuzzy permutation method with Student t test and with Wilcoxon rank sum test, and the Student t test without permutation. By contrast, the three tests with discretely distributed p-values (Wilcoxon rank sum test without permutation, and the standard permutation method with Student t test and with Wilcoxon rank sum test) are conservative, with the false discovery proportions being lower than the specified q-values, especially in the LS and LW scenarios. They are also very conservative in making significant calls, reporting not a single significant result in the LS scenario when the q-value is set below 0.1. The problem lies in their p values. First, any exact test strictly controlling the type I error rate is inherently conservative if its sampling distribution is discrete. And second, a key step in q-value computations is the estimation of the proportion of null hypotheses among the total variables tested^6^, but the estimation (which is based on the empirical distribution function of the p-values) will be more error-prone if the p-values themselves are discretely distributed.

**Figure 3:**
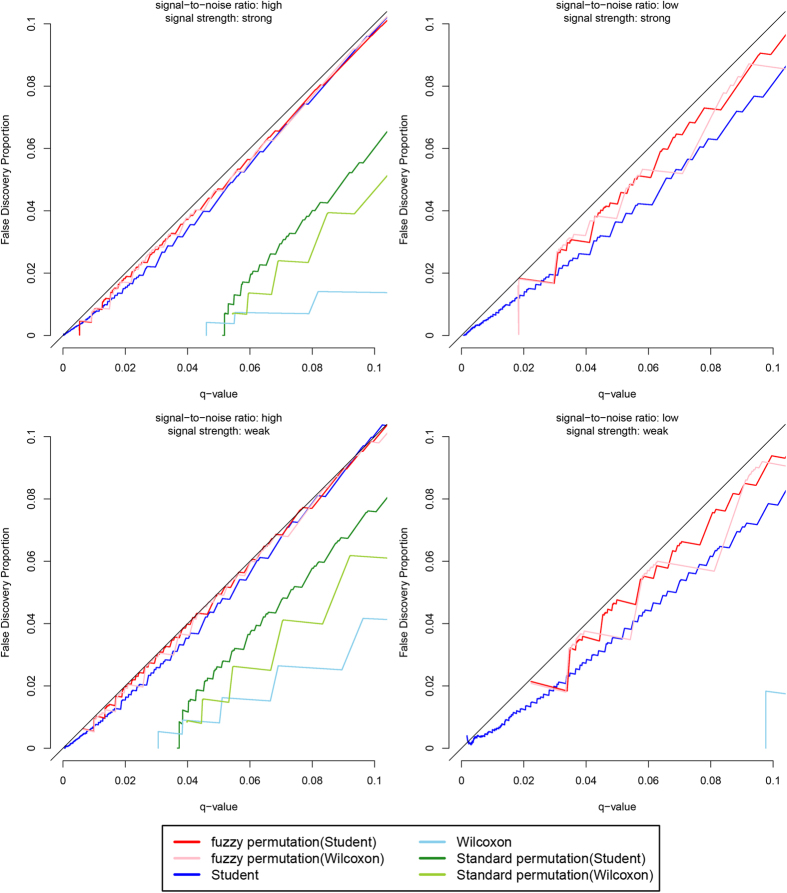
Plot of false discovery proportions against q-values, when the variables follow the gamma distribution.

**Figure 4:**
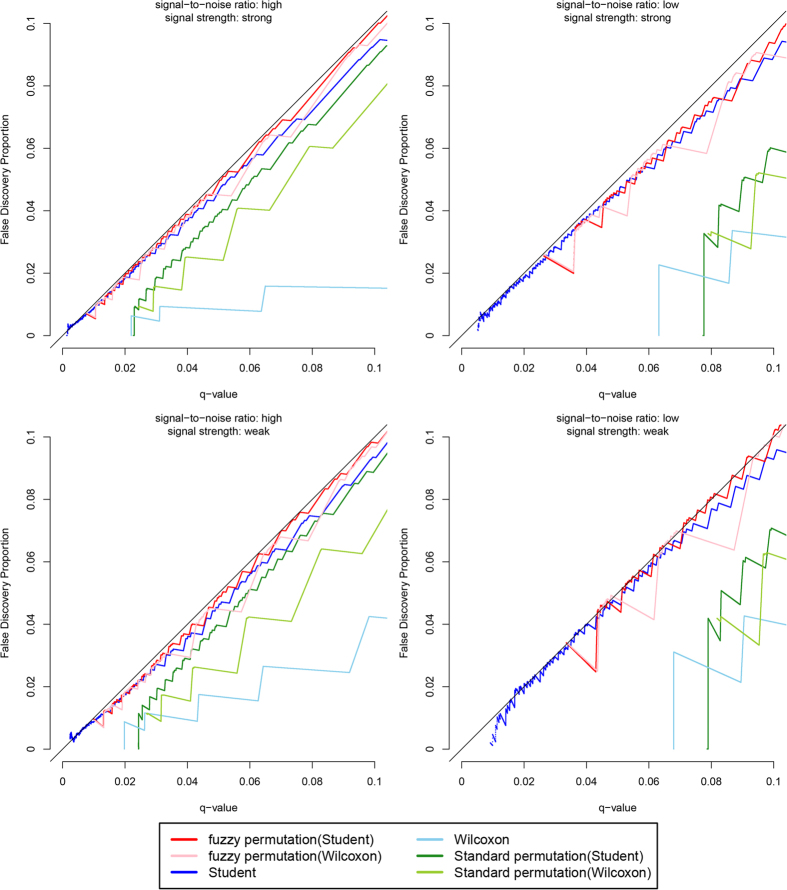
Plot of false discovery proportions against q-values, when the variables follow the truncated normal distribution.

Figure 3 shows the results when the variables follow the gamma distribution. In all four scenarios, the false discovery proportions are approximately equal to the specified q-values for the fuzzy permutation method with Student t test and with Wilcoxon rank sum test. Again, the three discrete tests are conservative for false discovery proportions and for significant calls, especially in the LS and LW scenarios. For the Student t test without permutation, the false discovery proportions are close to the specified q-values in the HS and HW scenarios, but is slightly lower than the q-values in the LS and LW scenarios. Figure 4 shows the results when the variables follow the truncated normal distribution. The findings are similar to those in Fig. 3.

**Figure 5:**
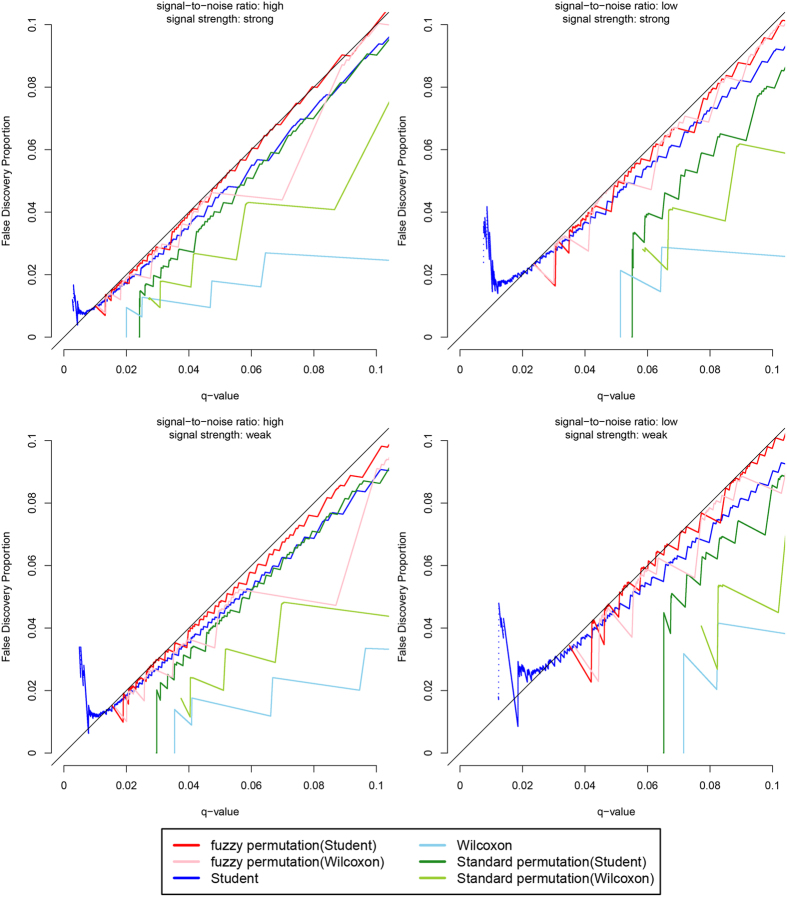
Plot of false discovery proportions against q-values, when the variables follow the beta distribution.

Figure 5 shows the results when the variables follow the beta distribution. The false discovery proportions for the Student t test without permutation are now greatly inflated, especially in the LS and LW scenarios. The false discovery proportion can soar up to more than 0.04 when the q-value is specified at 0.01. This means that the Student t test without permutation will report 30 more false discoveries than it is allowed to per 1000 discoveries it makes. The fuzzy permutation method with Student t test and with Wilcoxon rank sum test remain under control (false discovery proportions being approximately equal to or slightly lower than the specified q-values). As before, the three discrete tests are extremely conservative.

We reduced the total number of variables to 20000, and the results (S5–S8 Exhibits) are essentially the same as the above Figs 2, 3, 4, 5. (We did not further reduce the total number of variables, as Lin and Lee^12^ previously demonstrated that small total number of variables is associated with high variability of FDR controls—especially when we also take into account that the total number of subjects in this study is already very small). We increase the sample size to 60 (30 cases and 30 controls). All methods now produce similar results (S9–S12 Exhibits). This is as expected since now the central limit theorem applies.

### An Example

**Figure 6:**
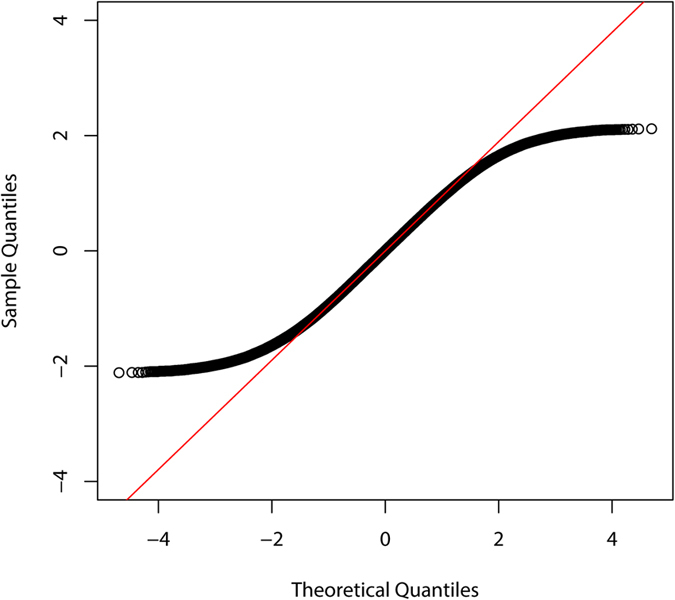
The Q-Q plot of the example data. The points represent sample quartiles and the straight line passes through the first and third quartiles of the standard normal distribution.

The gene expression data of Biswas and Poidinger^13^ is analyzed here for demonstration. The dataset consists of gene expression levels of a total of 47322 genes in blood monocytes samples from 4 renal cell carcinoma patients (the case group) and 4 healthy donors (the control group). The Q-Q plot (Fig. 6) shows that the distribution of gene expression levels is by no means normal; it has lighter tails than the standard normal distribution.

We use the six methods in the previous section to analyze this example. A total of 10000 permutations were performed. Table 1 shows that with FDR controlled at 0.05, none of the 47322 genes is significantly differentially expressed between the case and the control groups, except the Student t test without permutation. Under the FDR control value of 0.05, the Student t test without permutation detects a total of 5411 significant genes. This result, however, should not be taken for granted because the normality assumption fails in this example which is based on a sample size as small as 8. (Simulation study in the previous section has shown that with very small sample size, the conventional Student t test can yield inflated false discovery proportions if normality assumption fails.)

With FDR controlled at a higher value of 0.09, the fuzzy permutation method with Student t test can now detect a total of 245 significant genes. When FDR controlled at 0.10, the fuzzy permutation method with Student t test and with Wilcoxon rank sum test can even detect more significant genes than the Student t test without permutation.

For this example, the three discrete tests are extremely conservative in making significant calls. Not until FDR control value being raised to 0.20 that the standard permutation method with Student t test is beginning to detect 2 significant genes. When the FDR control value is raised to 0.40, the standard permutation method with Student t test can detect 18 significant genes, the Wilcoxon rank sum test without permutation, a total of 10523 significant genes, and the standard permutation method with Wilcoxon rank sum test, still zero significant gene. By contrast, with the same high FDR control value of 0.40, the fuzzy permutation method with Student t test can detect a total of 22745 significant genes, and the fuzzy permutation method with Wilcoxon rank sum test, a total of 22135 significant genes.

A study with a very small sample size (such as this example) should best be viewed as a screening study. Any statistically significant gene found in the study is to be subject to further confirmatory testing in studies with larger sample sizes. In consideration of this, many researchers may not opt for more significant genes in the initial screening study; due to limited budget they may work with only a fixed number of genes in subsequent follow-up large sample sized studies. Under this constraint, the fuzzy permutation method is still a better choice; the FDR control value can be set more stringent than other methods to obtain the same requested number of genes. For example, if one wishes to select for further study around 10000 genes from among the total 47322 genes in this example, the FDR control value for Wilcoxon rank sum test without permutation has to be set at 0.40, producing <10000 × 0.4 = 4000 false positives (this test was found to be conservative in the simulation study). But for the two fuzzy permutation methods, the FDR control value can be at 0.10, producing ~10000 × 0.1 = 1000 false positives (these two tests were found to be well controlled in the simulation study). If one wishes to select no more than 300 genes, the FDR control value for the standard permutation with Student t test will need to be set higher than 0.40 (with the number of false positives difficult to estimate), but for the fuzzy permutation with Student t test, it can be at 0.09 (with ~300 × 0.09 = 27 false positives).

## Discussion

Previously, Kulinskaya and Lewin also used randomized p-values for FDR control^14^. Their method is predicated on knowing the exact sampling distributions of the test statistics. Even if this is the case, the method has difficulty in determining the rejection regions for two-sided tests with non-symmetric sampling distributions. By contrast, our method uses an ingenious fuzzy permutation brute-force procedure to sidestep all the difficult mathematics and is applicable for all situations.

The standard permutation method^10^ is also a brute-force method for FDR control. It is completely data-driven without needing to specify the distributions in advance. In small sample size studies, ties are expected to be plentiful for any non-parametric (distribution-free) test based on ranks. Without the fuzzy device we created in this paper, the standard permutation method unfortunately cannot break a tie when it occurs. This will severely curtail its performances. In the simulation study, the standard permutation method was found to be extremely conservative. By contrast, the fuzzy permutation methods has desirable statistical properties as evidenced by our theoretical and simulational analysis.

In summary, the proposed method introduces fuzziness into standard permutation analysis to produce randomized p-values, which are then converted into q-values for FDR controls. It is a distribution-free method and is recommended for use in the large-p-small-n settings.

## Additional Information

**How to cite this article**: Yang, Y.-H. *et al*. A Fuzzy Permutation Method for False Discovery Rate Control. *Sci. Rep.* **6**, 28507; doi: 10.1038/srep28507 (2016).

## Supplementary Material

Supplementary Information

## Figures and Tables

**Table 1:** The number of significant genes for the example data.

Methods						
FDR* controlled at	fuzzy permutation with Student t test	fuzzy permutation with Wilcoxon rank sum test	Student t test without permutation	Wilcoxon rank sum test without permutation	standard permutation with Student t test	standard permutation with Wilcoxon rank sum test
0.05	0	0	5411	0	0	0
0.09	245	0	7737	0	0	0
0.10	9583	9557	8253	0	0	0
0.20	13885	13489	12691	0	2	0
0.40	22745	22135	22196	10523	18	0

^*^FDR: false discovery rate.
