# Supplementary material for: A Fuzzy Permutation Method for False Discovery Rate Control
Source: Sci Rep. 2016 Jun 22;6:28507. doi: 10.1038/srep28507 (PMC4916423; doi:10.1038/srep28507)
Supplement: Supplementary Information [file srep28507-s1.pdf]

Supplementary Information

accompanying the paper entitled

A Fuzzy Permutation Method for False Discovery Rate Control

by

Ya-Hui Yang, Wan-Yu Lin, and Wen-Chung Lee

S1 Exhibit. A permutation algorithm based on fractional counting.

S2 Exhibit. Theoretical comparisons of fuzzy and standard permutation methods.

S3 Exhibit. R code for fuzzy FDR controls.

S4 Exhibit. Parameters used in the simulation study.

S5 Exhibit. Simulation results for normal distribution (number of variables = 20000).

S6 Exhibit. Simulation results for gamma distribution (number of variables = 20000).

S7 Exhibit. Simulation results for truncated normal distribution (number of variables = 20000).

S8 Exhibit. Simulation results for beta distribution (number of variables = 20000).

S9 Exhibit. Simulation results for normal distribution (sample size = 60).

S10 Exhibit. Simulation results for gamma distribution (sample size = 60).

S11 Exhibit. Simulation results for truncated normal distribution (sample size = 60).

S12 Exhibit. Simulation results for beta distribution (sample size = 60).

S1 Exhibit. A permutation algorithm based on fractional counting

1. Perform a two-sample test, such as Student t test or Wilcoxon rank sum test, and calculate the p-value, p_j, for $j = 1,\ldots,m$.
2. Randomly select a fraction number f_j, for $j = 1,\ldots,m$, from U, a uniform (0,1) distribution.
3. Permute case and control status, and calculate the p-value $\tilde{p}_j$, for $j = 1,\ldots,m$, based on the permuted data.
4. Let $c_j = 1$ (full counting) if $\tilde{p}_j < p_j$, $c_j = f_j$ (fractional counting) if $\tilde{p}_j = p_j$, and $c_j = 0$ (no counting) if otherwise, for $j = 1,\ldots,m$.
5. Repeat Steps 3 and 4 for a total of k times.
6. Calculate a permutation-based p-value, p_j^{perm}, as the average counting among the total k permutations, for $j = 1,\ldots,m$.
7. Convert the permutation-based p-values in Step 6 to q-values.

S2 Exhibit. Theoretical comparisons of fuzzy and standard permutation methods

Let P denote a random variable, the realizations of which are p-values of a two-sample test regarding a specific variable in the permutations of a case-control study. Assume that P can take on at most L distinct values ($L \le C_{n_1}^{n_1+n_0}$, for a case-control study with n_1 cases and n_0 controls): $p_1, p_2, ..., p_L$ $(p_1 < p_2 < ... < p_L)$. Let $\Pr(P = p_i) = r_i$ for $i = 1, 2, ..., L$ $\left(\sum_{i=1}^{L} r_i = 1\right)$ be the probability mass distribution of P. Under the null hypothesis, the original data and each and every round of its permutation are equally likely to be observed. In other words, the original p-value is distributed the same as P.

Let P^{standard} denote the random variable of p-values derived from a standard permutation as the number of permutations approaches infinity. Under the null hypothesis, there is a probability of p_i that the p-value of the original data is the i th level of P. In that case, the standard permutation-based p-value will be $P^{\text{standard}} = s_i = \sum_{j=1}^{i} r_j$ (the probability that the p-value of the permuted data is at most as large as the original p-value). That is, P^{standard} has a probability mass distribution of $\Pr\left(P^{\text{standard}} = s_i\right) = r_i$ for $i = 1, 2, ..., L$ under the null hypothesis. Obviously, this is distributed as U, a uniform(0,1) distribution.

Let P^{fuzzy} denote the random variable of p-values derived from a fuzzy permutation (or equivalently the permutation algorithm based on fractional counting in S1 Exhibit) as the number of permutations approaches infinity. Under the null hypothesis, there is a probability of p_i that the p-value of the original data is the i th level of P. In that case, the fuzzy (fractional counting)

permutation-based p-value will be distributed as

s_{i-1} (from the full counting) + $r_i \times U$ (from the fractional counting), where we let $s_0 \equiv 0$. Taken together, P^{fuzzy} is a mixture of uniform distributions: a uniform (s_{i-1}, s_i) distribution with a probability of r_i, for $i=1, 2, ..., L$. Clearly, this amounts to a uniform (0,1) distribution.

Under an alternative hypothesis, the original p-value is not distributed the same as P. But it is still the case that when the p-value of the original data is the i th level of P, the standard permutation will report a permutation-based p-value as a point mass at s_i, and the fuzzy permutation will report a permutation-based p-value randomly sampled from a uniform (s_{i-1}, s_i) distribution. This indicates that algebraically, the fuzzy permutation-based p-value is at most as large as the standard permutation-based p-value. Therefore, the fuzzy permutation method is at least as powerful as the standard permutation method under any alternative.

S3 Exhibit. R code for fuzzy FDR controls.

```
##############################################################################
#    packages needed: doPrallel, doRNG, fdrtool                                              #
#    data: a (variables*samples) data matrix, with the 'cases' placed before the 'controls'   #
#    n.case: a scalar indicating the total number of 'cases' in the data                     #
#    n.perm: a scalar indicating the number of permutations (default value 10000)            #
##############################################################################

fuzz.perm <- function( data,    n.case,    n.perm=10000){
        epsilon <- 1e-6
        n1 <- n.case
        n <- ncol(data)
        n2 <- n - n1
        m <- nrow(data)
        n1_add_one <- n1 + 1
        data <- t(data)
        qval <- numeric(m)
        p <- apply(data,MARGIN=2,function(data)
                {t.test(data[1:n1],data[n1_add_one:n])$p.val})
        p_fuzz <- p + runif(m,0,epsilon)
        permu <- function(n,n1,data,p_fuzz){
                        cs <- sample(seq(1,n,1), n1, replace = F)
                        per.p <- apply(data, MARGIN=2,function(data)
                                {t.test(data[cs],data[-cs])$p.val})
                        per.p_fuzz <- per.p + runif(m,0,epsilon)
                        count <- as.numeric(per.p_fuzz <= p_fuzz)
                        return(count)
        }
        library (doParallel)
        cl <- makeCluster(28)
        registerDoParallel(cl)
        library(doRNG)
        set.seed(123)
        counts <- foreach(i=1:n.perm, .combine="+") %dorng% permu(n,n1,data,p_fuzz)
        stopCluster(cl)
        p_fuz_stud <- counts[1:m]/n.perm
        library(fdrtool)
```

```
    fd <- fdrtool(p_fuz_stud, statistic="pvalue")
    lfdr <- fd$lfdr
    sort.lfdr <- sort(lfdr, index.return = TRUE)
    for(i in 1:m){
            qval[i] <- mean(sort.lfdr$x[1:i])
    }
    return(qval)
}
```

S4 Exhibit. Parameters used in the simulation study.

The variables for the control subjects are generated using normal distribution ($\mu = 0, \sigma = 1$), gamma distribution (shape=4, scale=0.5), truncated normal distribution ($\mu = 0, \sigma = 1$, truncated at -1.5 and 1.5) and beta distribution ($\alpha = 0.5, \beta = 0.5$), respectively. The signal-to-noise ratios are 2.33 (H) and 0.67 (L) for normal distribution, 2.33 (H) and 0.67 (L) for gamma distribution, 2.33 (H) and 0.67 (L) for truncated normal distribution, and 2.33 (H) and 1.00 (L) for beta distribution, respectively. The signal strengths (difference in means between cases and controls) are 3.0 (S) and 2.0 (W) for normal distribution, 3.0 (S) and 2.5 (W) for gamma distribution, 1.8 (S) and 1.6 (W) for truncated normal distribution, and 0.8 (S) and 0.7 (W) for beta distribution, respectively.

S5 Exhibit. Simulation results for normal distribution (number of variables = 20000).

Parameters used in the simulation are the same as in the paper, except here the number of variables is 20000.

Figure Legends:

H and L: high and low signal-to-noise ratios;

S and W: strong and weak signals;

red: fuzzy permutation method with Student t test;

pink: fuzzy permutation method with Wilcoxon rank sum test;

blue: Student t test without permutation;

sky-blue: Wilcoxon rank sum test without permutation;

forest-green: standard permutation method with Student t test;

olive-drab: standard permutation method with Wilcoxon rank sum test.

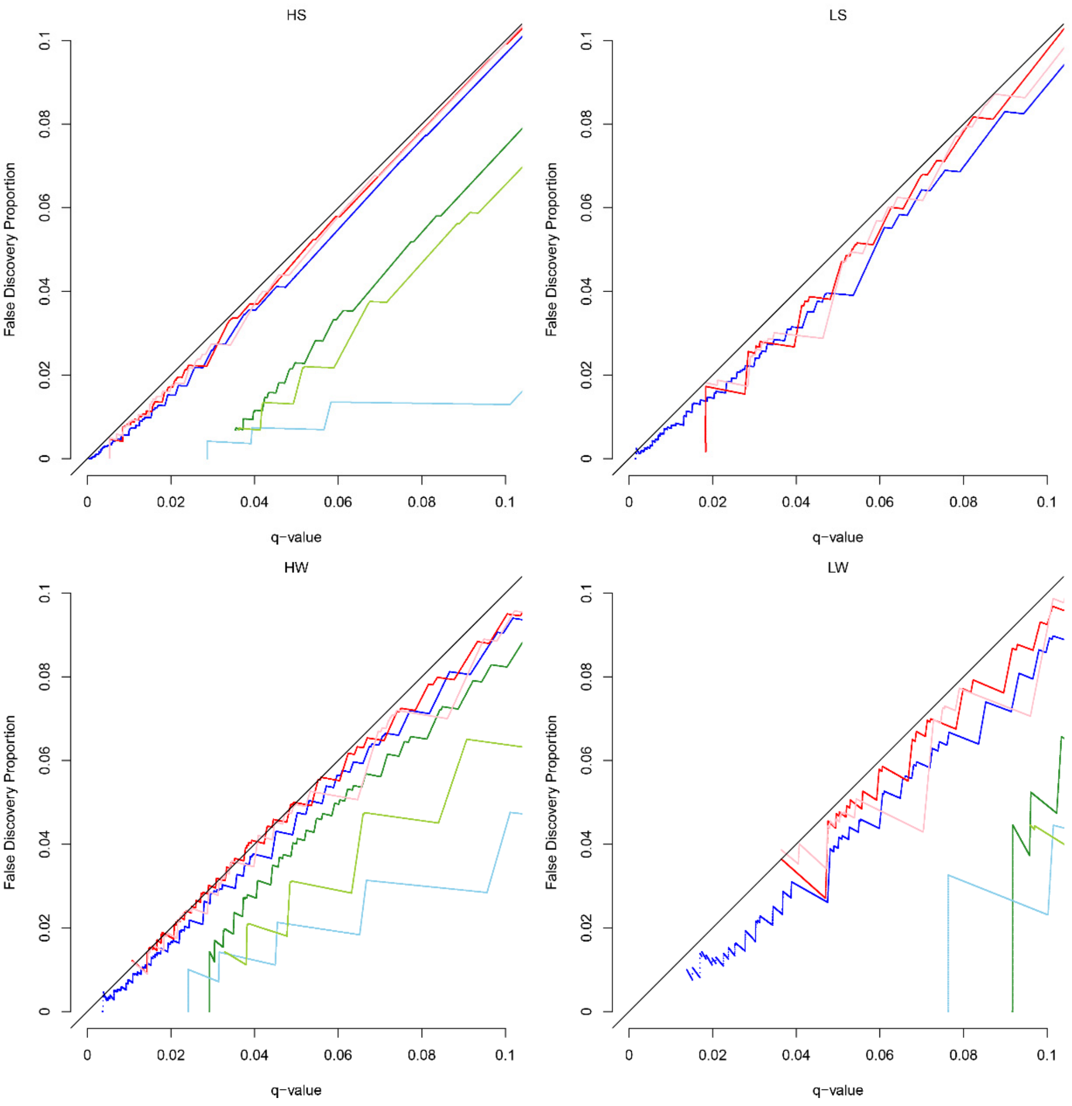
HS
LS
HW
LW
False Discovery Proportion
q−value
0
0.02
0.04
0.06
0.08
0.1

S6 Exhibit. Simulation results for gamma distribution (number of variables = 20000).

Parameters used in the simulation are the same as in the paper, except here the number of variables is 20000.

Figure Legends:

H and L: high and low signal-to-noise ratios;

S and W: strong and weak signals;

red: fuzzy permutation method with Student t test;

pink: fuzzy permutation method with Wilcoxon rank sum test;

blue: Student t test without permutation;

sky-blue: Wilcoxon rank sum test without permutation;

forest-green: standard permutation method with Student t test;

olive-drab: standard permutation method with Wilcoxon rank sum test.

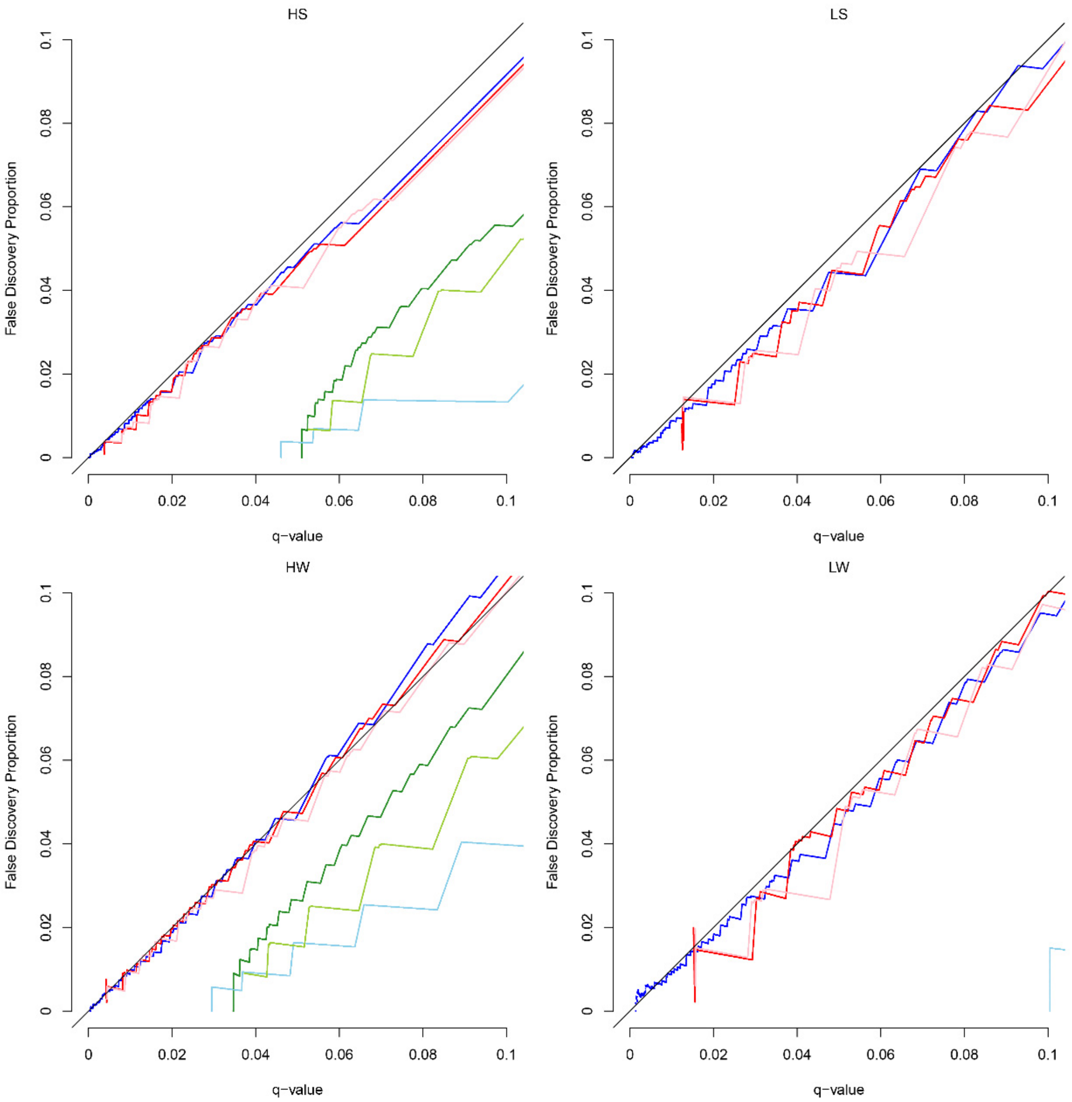
HS
LS
HW
LW
False Discovery Proportion
q−value
0
0.02
0.04
0.06
0.08
0.1

S7 Exhibit. Simulation results for truncated normal distribution (number of variables = 20000).

Parameters used in the simulation are the same as in the paper, except here the number of variables is 20000.

Figure Legends:

H and L: high and low signal-to-noise ratios;

S and W: strong and weak signals;

red: fuzzy permutation method with Student t test;

pink: fuzzy permutation method with Wilcoxon rank sum test;

blue: Student t test without permutation;

sky-blue: Wilcoxon rank sum test without permutation;

forest-green: standard permutation method with Student t test;

olive-drab: standard permutation method with Wilcoxon rank sum test.

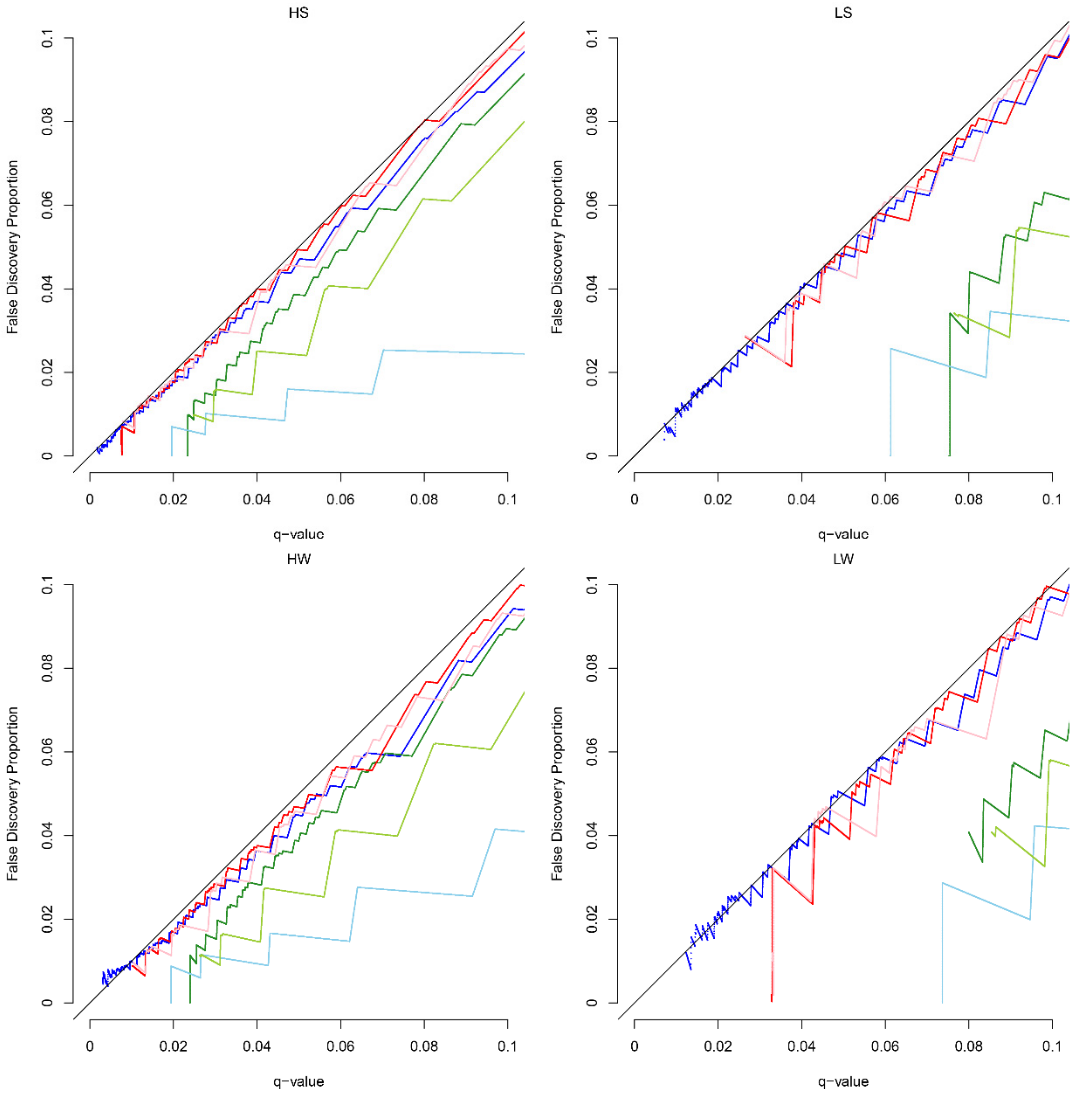
HS
LS
HW
LW
False Discovery Proportion
q−value
0
0.02
0.04
0.06
0.08
0.1

S8 Exhibit. Simulation results for beta distribution (number of variables = 20000).

Parameters used in the simulation are the same as in the paper, except here the number of variables is 20000.

Figure Legends:

H and L: high and low signal-to-noise ratios;

S and W: strong and weak signals;

red: fuzzy permutation method with Student t test;

pink: fuzzy permutation method with Wilcoxon rank sum test;

blue: Student t test without permutation;

sky-blue: Wilcoxon rank sum test without permutation;

forest-green: standard permutation method with Student t test;

olive-drab: standard permutation method with Wilcoxon rank sum test.

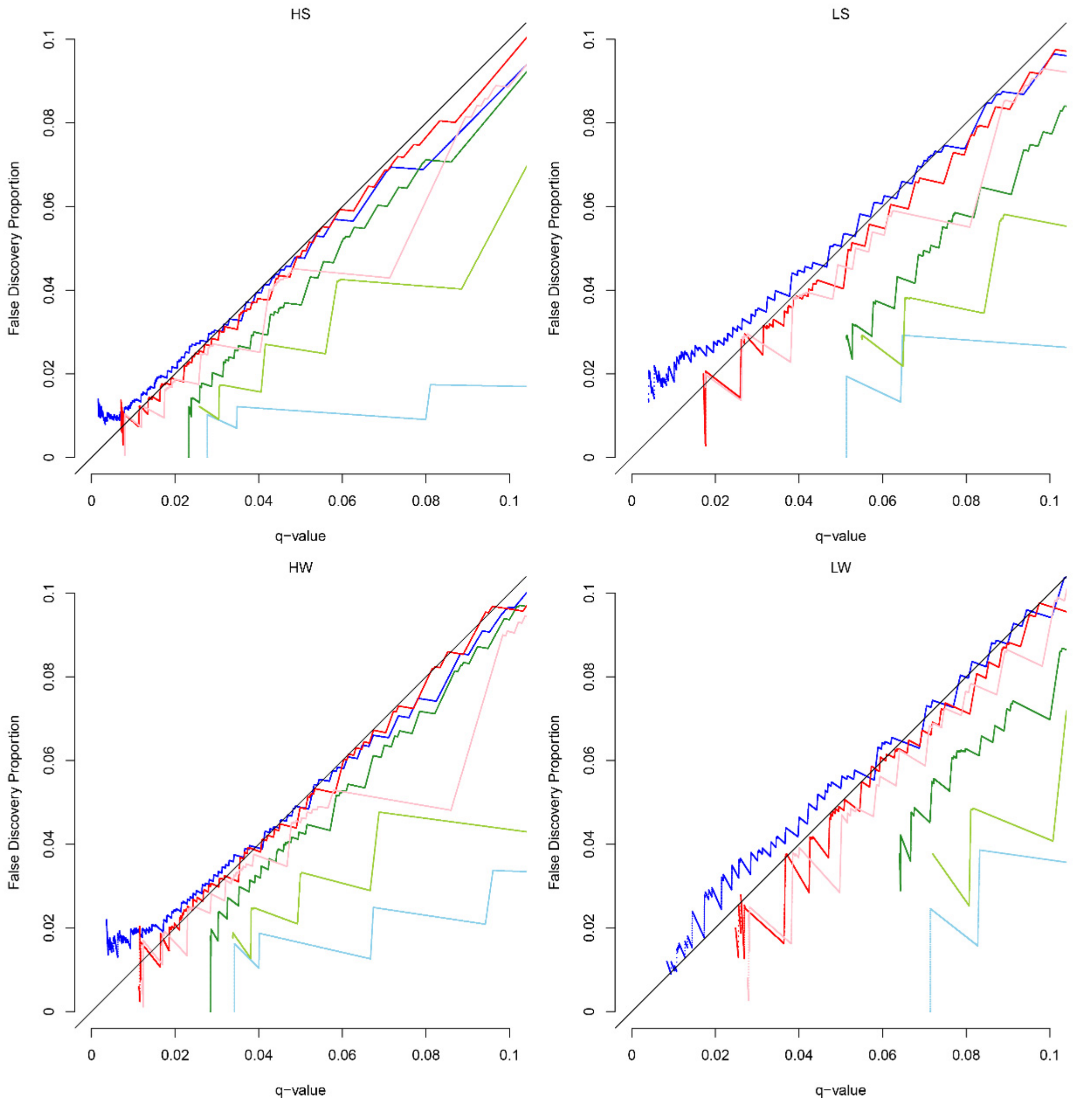
HS
LS
HW
LW
False Discovery Proportion
q−value
0
0.02
0.04
0.06
0.08
0.1

S9 Exhibit. Simulation results for normal distribution (sample size=60).

The variables for the control subjects are generated using the standard normal distribution. The total number of variables is 100000. The sample size is 60 (30 cases and 30 controls). A total of 5000 permutations were performed. The signal-to-noise ratios are 2.33 (H) and 0.67 (L). The signal strengths (difference in means between cases and controls) are 1.095 (S) and 0.73 (W).

Figure Legends:

H and L: high and low signal-to-noise ratios;

S and W: strong and weak signals;

red: fuzzy permutation method with Student t test;

pink: fuzzy permutation method with Wilcoxon rank sum test;

blue: Student t test without permutation;

sky-blue: Wilcoxon rank sum test without permutation;

forest-green: standard permutation method with Student t test;

olive-drab: standard permutation method with Wilcoxon rank sum test.

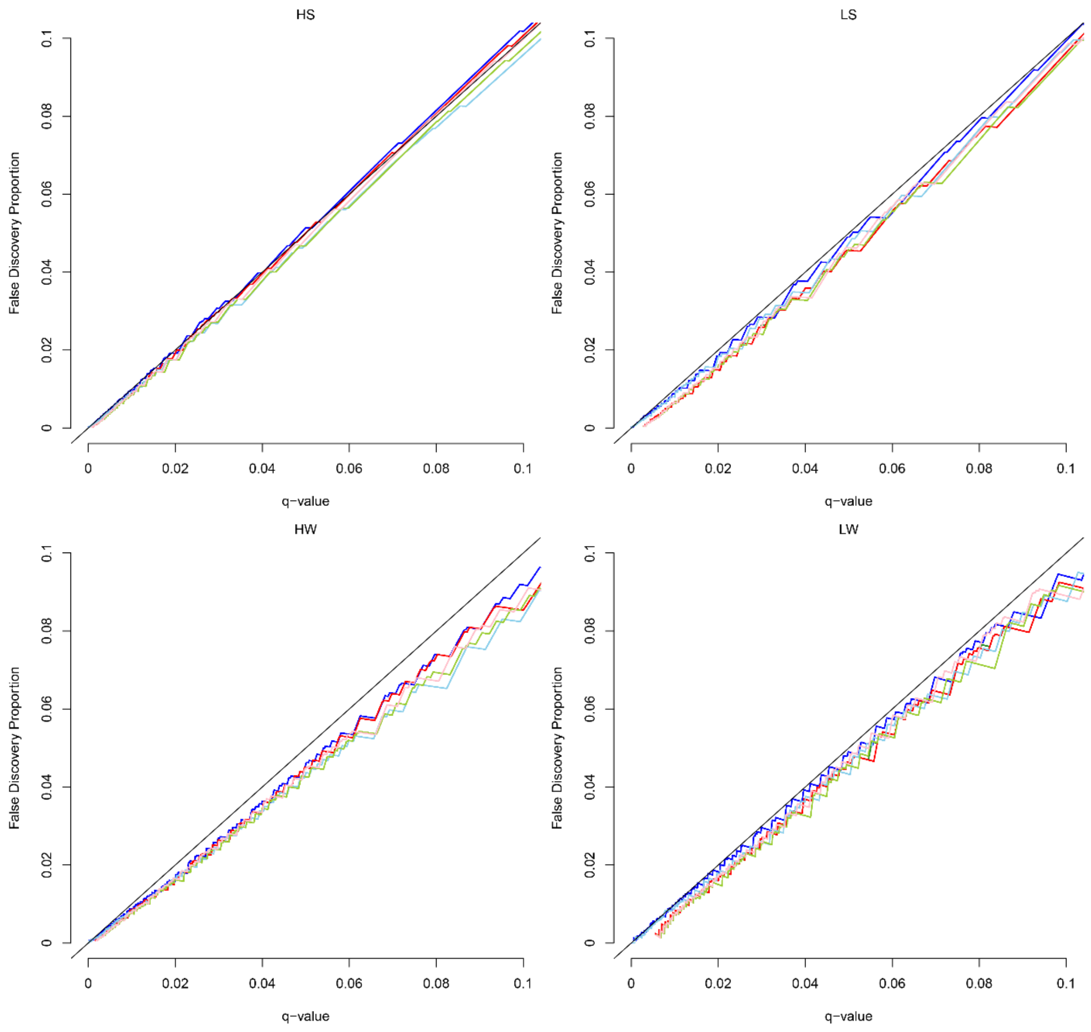
HS
LS
HW
LW
False Discovery Proportion
q−value
0
0.02
0.04
0.06
0.08
0.1

S10 Exhibit. Simulation results for gamma distribution (sample size=60).

The variables for the control subjects are generated using the gamma distribution (shape=4, scale=0.5). The total number of variables is 100000. The sample size is 60 (30 cases and 30 controls). A total of 5000 permutations were performed. The signal-to-noise ratios are 2.33 (H) and 0.67 (L). The signal strengths (difference in means between cases and controls) are 1.3 (S) and 1.1 (W).

Figure Legends:

H and L: high and low signal-to-noise ratios;

S and W: strong and weak signals;

red: fuzzy permutation method with Student t test;

pink: fuzzy permutation method with Wilcoxon rank sum test;

blue: Student t test without permutation;

sky-blue: Wilcoxon rank sum test without permutation;

forest-green: standard permutation method with Student t test;

olive-drab: standard permutation method with Wilcoxon rank sum test.

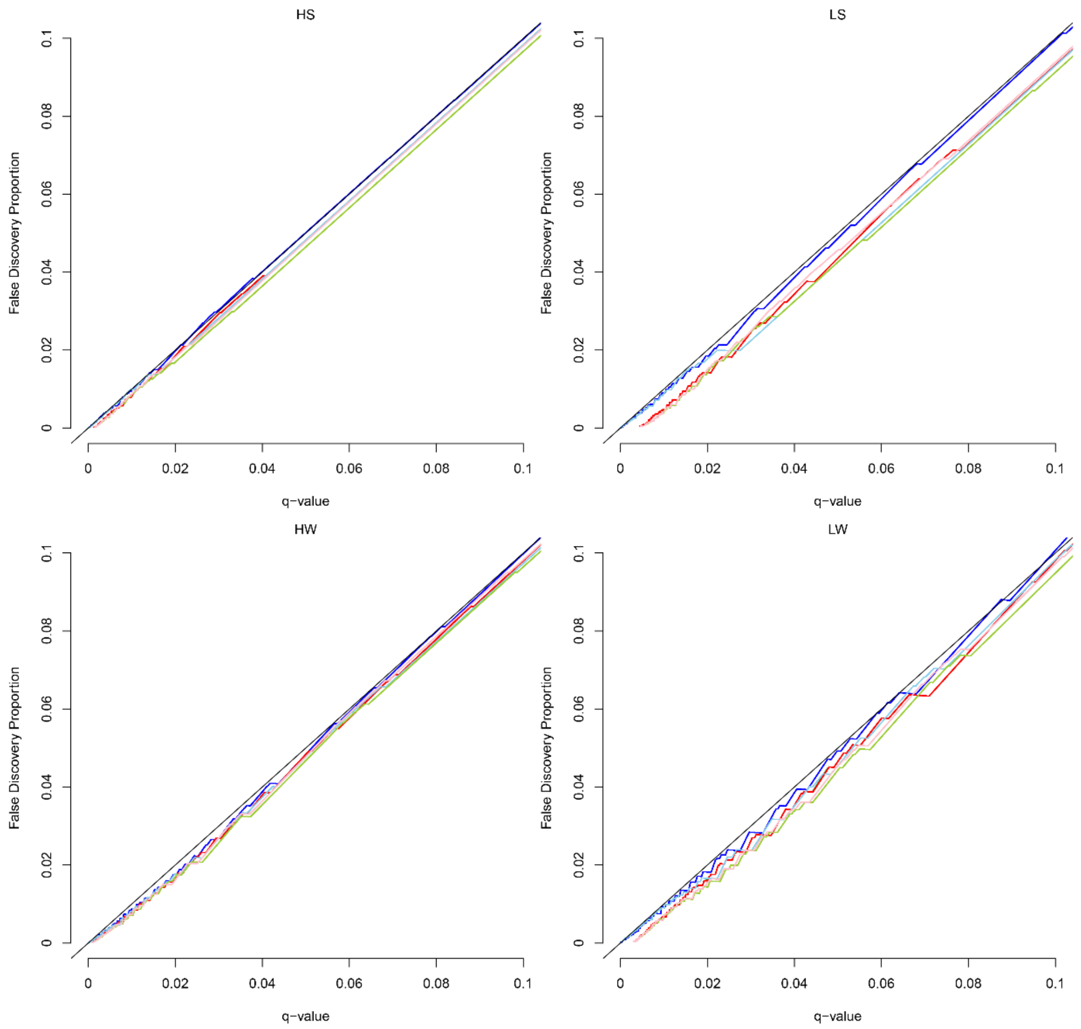
HS
LS
HW
LW
False Discovery Proportion
q−value
0
0.02
0.04
0.06
0.08
0.1

S11 Exhibit. Simulation results for truncated normal distribution (sample size = 60).

The variables for the control subjects are generated using the truncated normal distribution ($\mu = 0, \sigma = 1$, truncated at -1.5 and 1.5). The total number of variables is 100000. The sample size is 60 (30 cases and 30 controls). A total of 5000 permutations were performed. The signal-to-noise ratios are 2.33 (H) and 0.67 (L). The signal strengths (difference in means between cases and controls) are 1.3 (S) and 1.1 (W).

Figure Legends:

H and L: high and low signal-to-noise ratios;

S and W: strong and weak signals;

red: fuzzy permutation method with Student t test;

pink: fuzzy permutation method with Wilcoxon rank sum test;

blue: Student t test without permutation;

sky-blue: Wilcoxon rank sum test without permutation;

forest-green: standard permutation method with Student t test;

olive-drab: standard permutation method with Wilcoxon rank sum test.

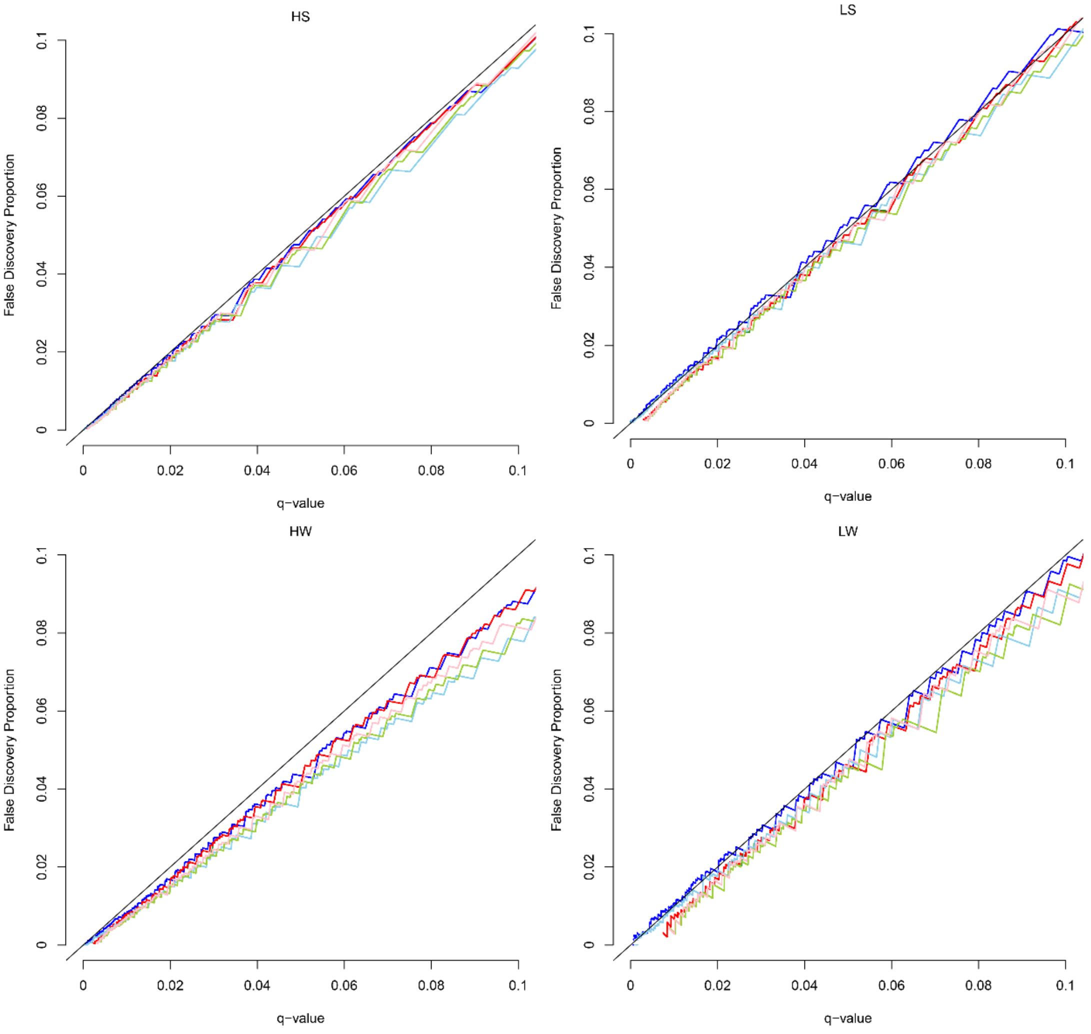
HS
LS
HW
LW
False Discovery Proportion
q−value
0
0.02
0.04
0.06
0.08
0.1

S12 Exhibit. Simulation results for beta distribution (sample size=60).

The variables for the control subjects are generated using the beta distribution ($\alpha = 0.5, \beta = 0.5$). The total number of variables is 100000. The sample size is 60 (30 cases and 30 controls). A total of 5000 permutations were performed. The signal-to-noise ratios are 2.33 (H) and 1 (L). The signal strengths (difference in means between cases and controls) are 0.5 (S) and 0.4 (W).

Figure Legends:

H and L: high and low signal-to-noise ratios;

S and W: strong and weak signals;

red: fuzzy permutation method with Student t test;

pink: fuzzy permutation method with Wilcoxon rank sum test;

blue: Student t test without permutation;

sky-blue: Wilcoxon rank sum test without permutation;

forest-green: standard permutation method with Student t test;

olive-drab: standard permutation method with Wilcoxon rank sum test.

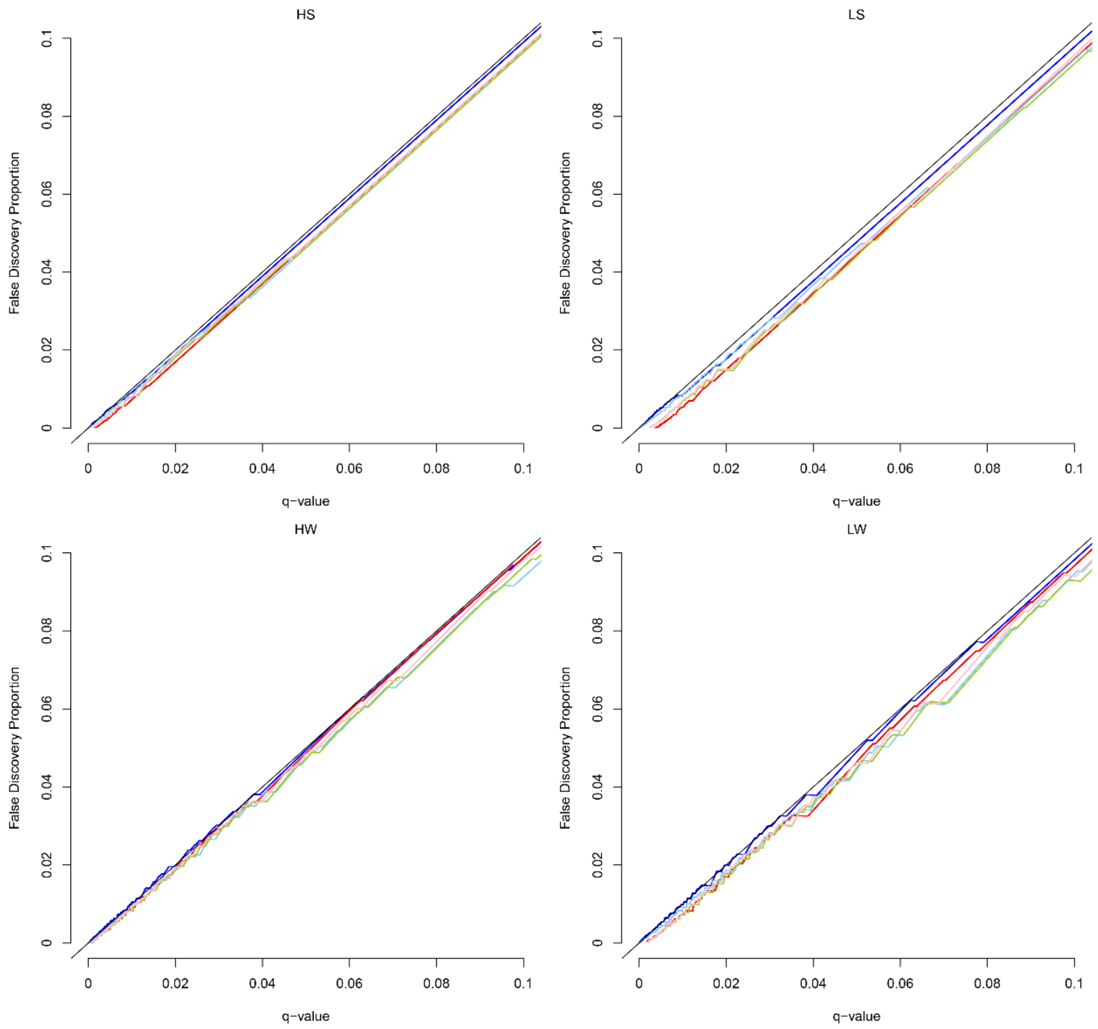
HS
LS
HW
LW
False Discovery Proportion
q−value
0
0.02
0.04
0.06
0.08
0.1
